# Marine Alkaloid 2,2-Bis(6-bromo-3-indolyl) Ethylamine and Its Synthetic Derivatives Inhibit Microbial Biofilms Formation and Disaggregate Developed Biofilms

**DOI:** 10.3390/microorganisms7020028

**Published:** 2019-01-23

**Authors:** Raffaella Campana, Gianfranco Favi, Wally Baffone, Simone Lucarini

**Affiliations:** 1 Department of Biomolecular Science, Division of Toxicological, Hygiene and Environmental Science, Via S. Chiara 27, University of Urbino Carlo Bo, 61029 Urbino, Italy; wally.baffone@uniurb.it; 2 Department of Biomolecular Science, Section of Organic Chemistry and Organic Natural Compounds, University of Urbino Carlo Bo, Via I Maggetti 24, 61029 Urbino, Italy; gianfranco.favi@uniurb.it; 3 Department of Biomolecular Science, Division of Chemistry, Piazza del Rinascimento 6, University of Urbino Carlo Bo, 61029 Urbino, Italy

**Keywords:** marine bisindole alkaloids, bisindole derivatives, anti-biofilm activity

## Abstract

The antimicrobial activity of the marine bisindole alkaloid 2,2-bis(6-bromo-3-indolyl) ethylamine (**1**) and related synthetic analogues (compounds **2**–**8**) against target microorganisms was investigated by Minimum Inhibitory Concentration (MIC) determination. Compound **1** showed the greatest antimicrobial activity with the lowest MIC (8 mg/L) against *Escherichia coli*, *Staphylococcus aureus*, and *Klebsiella pneumoniae*, while the derivatives exhibited higher MICs values (from 16 to 128 mg/L). Compounds **1**, **3**, **4**, and **8**, the most active ones, were then tested against *E. coli*, *S. aureus*, *K. pneumoniae*, and *Candida albicans* during biofilms formation as well as on 24 h developed biofilms. The natural alkaloid **1** inhibited the biofilm formation of all the tested microorganisms up to 82.2% and disaggregated biofilms of *E. coli*, *S. aureus*, *K. pneumoniae*, and *C. albicans* after 30 min of contact, as assessed by viable plate count and crystal violet (CV) staining (optical density at 570 nm). Synthetic derivatives **3**, **4**, and **8** displayed anti-biofilm activity toward individual bacterial populations. This study highlights the potential of marine bisindole alkaloid **1** as anti-biofilm agent and shows, through a preliminary structure activity relationship (SAR), the importance of halogens and ethylamine side chain for the antimicrobial and antibiofilm activities of this bisindole series.

## 1. Introduction

Biofilms represent the predominant phenotype of most bacteria in their natural habitat. The biofilm formation requires a first phase of adhesion to the surface, after the cells start to replicate into micro-colonies and to produce an extracellular polymeric substance (EPS), composed of polysaccharides and other macromolecules [1,2]. The EPS plays an important role in biofilm because it helps the bond between the bacteria and the substratum and it protects the colonies from any environmental stress, including antimicrobial treatment [3]. Food surfaces as well as medical environments are suitable surfaces for microbial colonization and subsequently biofilm formation [4], thus representing a potential risk to transmit pathogens to humans by cross-contaminations. Moreover, biofilms that were developed on medical devices are difficult to eradicate due to the significant decrease in the susceptibility to antimicrobials of bacteria organized in biofilm, as compared to their planktonic form [5].

**Figure 1:**
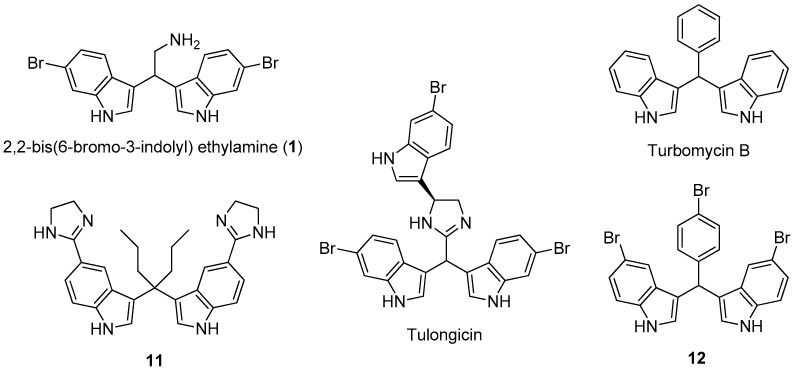
The marine alkaloid 1, some natural and synthetic antibacterial agents having the 3,3’-diindolylmethane (DIM) molecular unit.

Marine natural products show potent activity and selectivity against a wide spectrum of pharmacological targets and their unprecedented structures are often an important source of lead compounds in drug discovery and development [6]. Among the various structural classes, the marine bisindole alkaloids, dimers of indole typically produced by marine sponges [7,8], have received much attention due to their significant cytotoxicity, antineoplastic activity, as well as antimicrobial and antibiofilm activity [9,10,11]. Notably, 2,2-bis(6-bromo-3-indolyl)ethylamine (compound **1**) (Figure 1), isolated from the Californian tunicate *Didemnum candidum* and the New Caledonian sponge *Orina* spp. [12,13], has shown antiplasmodial activity [14,15], cytotoxicity against several tumor cell lines, such as U937 human leukemia [16,17], MCF-7 human breast, and Caco-2 human epithelial colorectal [18]. Several natural and synthetic products with antibacterial and antibiofilm activity (tulongicin, turbomycin B, etc.) (Figure 1) share with compound **1**, a common 3,3’-diindolylmethane (DIM) molecular unit [19,20,21].

For example, tulongicin, derived from a *Topsentia* sp. marine sponge, is a bisindolemethane linked to an imidazole group that inhibited the growth of *S. aureus* [22]. Compound **11**, which was developed by Dong and co-workers [23], showed to be very potent against several methicillin-resistant *S. aureus* clinical strains both in the planktonic and biofilm forms. However, to the best of our knowledge, no studies were devoted to the antimicrobial and antibiofilm abilities of **1** and analogues. Moreover, the presence of the alkylamino side chain in the marine alkaloid **1** could improve its solubility in water, possibly enhancing the antimicrobial and/or antibiofilm activity and allowing the possibility of further synthetic transformations by amino derivatization and/or conjugation. 

**Figure 2:**
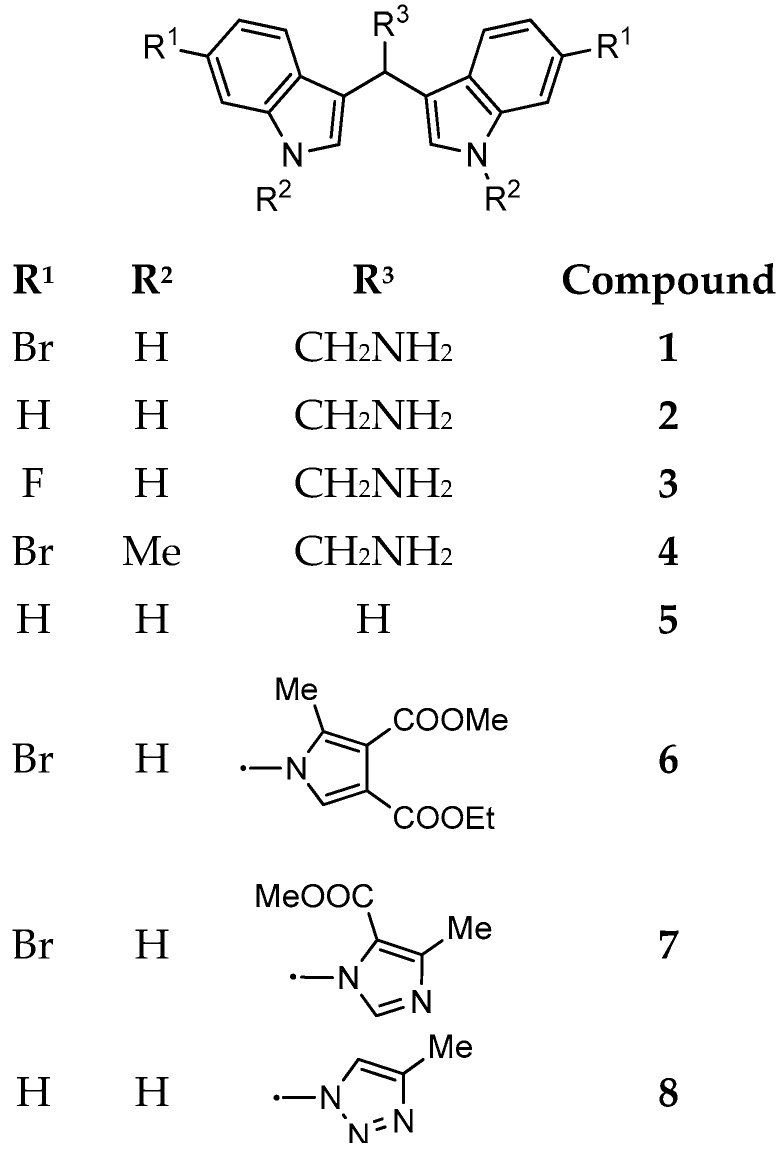
Marine bisindole alkaloid **1** and its synthetic derivatives **2**–**8**.

For these reasons, in the present study, the marine bisindole alkaloid **1** and some related derivatives were synthesized (Figure 2) and tested against several target microorganisms, including Gram-postitive, Gram-negative, and mycetes, by Minimum Inhibitory Concentration (MIC) determination. The most active compounds were then tested for their anti-biofilm activity during biofilms formation as well as on developed biofilms. 

## 2. Materials and Methods

### 2.1. Chemicals

Bis(1H-indol-3-yl)methane (compound **5**) and all organic solvents that were used in this study were purchased from Sigma (Milan, Italy). Prior to use, acetonitrile was dried with molecular sieves with an effective pore diameter of 4 Å. Column chromatography purifications were performed under ‘‘flash” conditions using Merck 230–400 mesh silica gel. Analytical thin-layer chromatography (TLC) was carried out on Merck silica gel plates (silica gel 60 F254), which were visualized by exposure to ultraviolet light and an aqueous solution of cerium ammonium molybdate (CAM). ESI-MS spectra were recorded with a Waters Micromass ZQ spectrometer). EI-MS spectra were recorded with a Shimadzu QP-5000 Mass spectrometer. ^1^H NMR and ^13^C NMR spectra were recorded on a Bruker AC 400 or 100, respectively, spectrometer and analyzed using the TopSpin software package. Chemical shifts were measured by using the central peak of the solvent.

### 2.2. Chemistry

**Figure 3:**
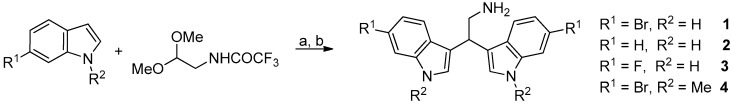
Reaction conditions: (**a**) diphenyl phosphate, acetonitrile, 80 °C, 24 h; (**b**) K_2_CO_3_, methanol, reflux, 2 h.

Marine bisindole alkaloid **1** and compounds **2**–**4** were prepared, as described in Figure 3.

#### 2.2.1. General Procedure for the Synthesis of Derivatives **1**–**4**

Diphenyl phosphate (0.02 mmol) was added to a solution of the appropriate indole derivative (0.4 mmol) and (trifluoroacetylamino)acetaldehyde dimethyl acetal (0.2 mmol) in anhydrous acetonitrile (0.2 mL), and the resulting mixture was stirred at 80 °C for 24 h in a sealed tube, monitoring the progress of the reaction by TLC and HPLC-MS. After cooling to room temperature, saturated aqueous NaHCO_3_ (30 mL) and dichloromethane (30 mL) were added and the two phases were then separated. The aqueous solution was extracted with dichloromethane (3 × 20 mL). After drying over dry Na_2_SO_4_, the combined organic phases were concentrated in vacuum and the resulting crude product was utilized without further purification. A mixture of that crude trifluoroacetamide derivative and potassium carbonate (1 mmol) in MeOH (1.87 mL) and H_2_O (0.13 mL) was stirred and heated at reflux for 2 h. The MeOH was removed under reduced pressure and water was added (30 mL). The aqueous solution was extracted with dichloromethane (3 × 30 mL) and the resulting solution was dried with Na_2_SO_4_ and then concentrated in vacuum. The crude material was purified by flash chromatography on neutral alumina.

#### 2.2.2. 2,2-Bis(6-Bromo-1*H*-Indol-3-Yl) Ethylamine (**1**) and 2,2-Bis(1*H*-Indol-3-Yl) Ethylamine (**2**)

The physico-chemical data of compounds **1** and **2** are in agreement with those that were reported [17].

#### 2.2.3. 2,2-Bis(6-Fluoro-1*H*-Indol-3-Yl) Ethylamine (**3**)

Compound **3** was prepared employing 6-fluoro-1*H*-indole and was isolated by column chromatography (dichloromethane/methanol/triethylamine, 90:9:1) as a white solid in 70% yield (two steps). TLC: Rf = 0.18 (silica gel; dichloromethane/ methanol/ triethylamine, 90:9:1; UV, CAM). MS (ESI): *m*/*z* 310 [M-H]^−^. ^1^H NMR (400 MHz, CD_3_OD, 293 K): δ = 3.37-3.41 (m, 2H, CHC*H_2_*NH_2_), 4.52 (dd, 1H, *J*_1_ = *J*_2_ = 7.5 Hz, C*H*CH_2_NH_2_), 6.72 (ddd, 2H, *J*_5-7_ = 2.0 Hz, *J*_5-4_ = 9.0 Hz, *J*_5-F_ = 9.5 Hz, H5), 7.04 (dd, 2H, *J*_7-5_ = 2.0 Hz, *J*_7-F_ = 9.5 Hz, H7), 7.14 (d, 2H, *J* = 3.0 Hz, H2), 7.44 (dd, 2H, *J*_4-F_ = 5.0 Hz, *J*_4-5_ = 9.0 Hz, H4) ppm. ^13^C NMR (100 MHz, CD_3_OD, 293 K): δ = 37.2, 45.6, 96.7 (d, 2C, *J* = 26 Hz, C5), 106.5 (d, 2C, *J* = 24 Hz, C7), 116.4 (2C, C3), 119.5 (d, 2C, *J* = 10 Hz, C4), 122.3 (d, 2C, *J* = 3 Hz, C9), 123.6 (2C, C2), 137.0 (d, 2C, *J* = 12 Hz, C8), 159.7 (d, 2C, *J* = 233 Hz, C6) ppm.

#### 2.2.4. 2,2-Bis(6-Bromo-1-Methyl-1*H*-Indol-3-Yl) Ethylamine (**4**)

Compound **4** was prepared employing 6-bromo-1-methylindole and it was isolated by column chromatography (dichloromethane/ methanol/ triethylamine, 90:9:1) as yellowish solid (foam) in 77% yield (two steps). TLC: Rf = 0.22 (silica gel; dichloromethane/ methanol/ triethylamine, 90:9:1; UV, CAM). MS (ESI): m/z 460 (50), 462 (100), 464 (50) [M + H]^+^. ^1^H NMR (400 MHz, CD_3_OD, 293 K): δ = 3.32 (d, 2H, *J* = 7.5 Hz, CHC*H_2_*NH_2_), 3.72 (s, 6H, NC*H_3_*), 4.49 (dd, 1H, *J*_1_ = *J*_2_ = 7.5 Hz, C*H*CH_2_NH_2_), 7.06 (s, 2H, ArH), 7.06 (dd, 2H, *J*_1_ = 1.5 Hz, *J*_2_ = 8.5 Hz, ArH), 7.40 (d, 2H, *J* = 8.5 Hz, ArH), 7.51 (d, 2H, *J* = 1.5 Hz, ArH) ppm. ^13^C NMR (100 MHz, CD_3_OD, 293 K): δ = 31.4, 36.8, 45.8, 112.0, 114.7, 115.9, 120.2, 121.4, 126.3, 127.2, 138.2 ppm.

**Figure 4:**
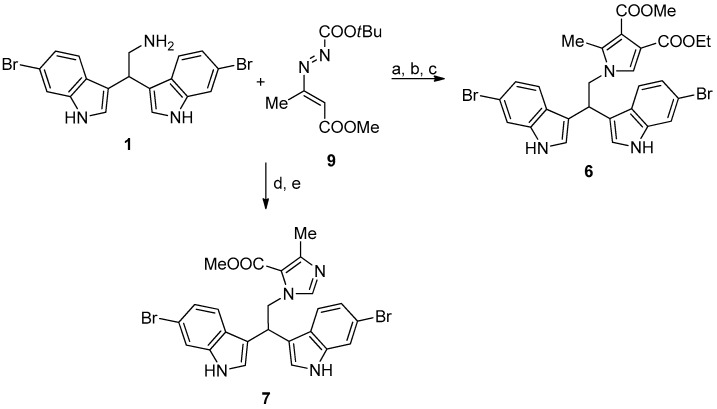
Reaction conditions: (**a**) ethyl propiolate, dichloromethane, at room temperature (RT), overnight (ON); (**b**) **9**, toluene, reflux, 2 h; (**c**) trifluoroacetic acid, reflux, 4h. (**d**) **9**, acetonitrile, RT, 1h; and, (**e**) paraformaldehyde, reflux, 4h.

Compounds **6** and **7** were synthesized, as reported in Figure 4.

#### 2.2.5. 4-Ethyl 3-Methyl 1-(2,2-Bis(6-Bromo-1*H*-Indol-3-Yl)Ethyl)-2-Methyl-1*H*-Pyrrole-3,4-Dicarboxylate (**6**)

A mixture of **1** (0.4 mmol) and ethyl propiolate (0.44 mmol) was stirred in dichloromethane (1 mL) overnight at room temperature. A solution of **9** (0.6 mmol) in toluene (4 mL) was added and the reaction was refluxed for 2 h. Catalytic amount of trifluoroacetic acid (two drops) was added and the reaction was refluxed for additional 4 h. After removal of the solvent, the crude mixture was purified by column chromatography on silica gel (ethyl acetate/cyclohexane 25:75) to afford product **6** in 70% yield as a yellow solid. MS (EI) *m/z* (%) = 627 (M^+^) (3), 405 (31), 403 (100), 401 (38). ^1^H NMR (400 MHz, DMSO-d6, 293 K): δ = 1.17 (t, 3H, *J* = 7.2 Hz, OCH_2_C*H_3_*), 2.11 (s, 3H, CH_3_), 3.64 (s, 3H, OCH_3_), 4.06 (q, *J* = 7.2 Hz, 2 H, OC*H_2_*CH_3_), 4.63 (d, 2H, *J* = 8.0 Hz, CHC*H_2_*N), 4.87 (t, 1H, *J* = 8.0 Hz, C*H*CH_2_N), 6.99 (dd, 2H, *J*_1_ = 1.6 Hz, *J*_2_ = 8.8 Hz, ArH), 7.16 (s, 1H, ArH), 7.37−7.42 (m, 4H, ArH), 7.49 (d, 2H, *J* = 1.6 Hz, Ar-H), 11.06 (brs, 2H, NH). ^13^C NMR (100 MHz, DMSO-d6, 293 K): δ = 10.7, 14.6, 35.2, 50.9, 51.4, 59.7, 100.0, 114.0, 114.2, 114.4, 115.4, 121.0, 121.6, 124.4, 125.8, 127.5, 135.3, 137.6, 163.7, 165.6.

#### 2.2.6. Methyl 1-(2,2-Bis(6-Bromo-1*H*-Indol-3-Yl)Ethyl)-4-Ethyl-1*H*-Imidazole-5-Carboxylate (**7**)

To a stirred solution of **1** (0.4 mmol) in acetonitrile (2 mL), **9** (0.4 mmol) was added at room temperature. After the disappearance of the reagents, paraformaldehyde (0.8 mmol) was added, and then the resulting mixture was refluxed for 4 h (TLC check). The solvent was evaporated under reduced pressure and the crude residue was purified by column chromatography (ethyl acetate/cyclohexane 80:20) to give **7** as a brown solid in 72% yield. MS (EI) *m/z* (%) = 556 (M^+^) (100), 401 (32), 403 (100), 405 (39). ^1^H NMR (400 MHz, DMSO-d6, 293 K): δ = 2.26 (s, 3H, CH_3_), 3.80 (s, 3H, OCH_3_), 4.91–4.96 (m, 3H, C*H*C*H_2_*N), 7.01 (dd, 2H, *J_1_* = 1.6 Hz, *J_2_* = 8.4 Hz, ArH), 7.22 (s, 1H, ArH), 7.30 (d, 2H, *J* = 2.0 Hz, ArH), 7.40 (d, 2H, *J* = 8.4 Hz, ArH), 7.50 (d, 2H, *J* = 1.6 Hz, ArH), 11.04 (brs, 2H, NH). ^13^C NMR (100 MHz, DMSO-d6, 293 K): δ = 16.1, 35.4, 51.0, 51.8, 114.2, 114.5, 115.4, 117.9, 120.8, 121.6, 124.4, 125.9, 137.6, 142.2, 147.4, 161.5.

**Figure 5:**
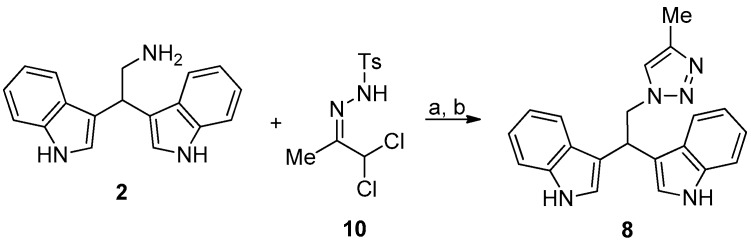
Reaction conditions: (**a**) *N*,*N*-diisopropylethylamine, ethanol, 0 °C, 10 min; and, (**b**) **10**, acetonitrile, 0 °C, RT, 2 h.

Compound **8** was prepared as reported in Figure 5.

#### 2.2.7. 3,3′-(2-(4-Methyl-1*H*-1,2,3-Triazol-1-Yl)Ethane-1,1-Diyl)Bis(1*H*-Indole) (**8**)

To a cooled solution (0 °C) of **2** (0.4 mmol) in ethanol (5 mL) was added *N*,*N*-diisopropylethylamine (2.4 mmol, 6 eq). The solution was stirred for 10 min. after which hydrazone **10** (0.52 mmol, 1.3 eq.) dissolved in acetonitrile (4 mL) was added dropwise to the cooled solution and stirring was continued at room temperature until completion of the reaction (TLC check). After completion of the reaction, all volatiles were removed under reduced pressure and the residue was purified by column chromatography (ethyl acetate/cyclohexane 60:40) to give **8** as pale yellow solid in quantitative yield.

MS (EI) *m/z* (%) = 341 (M^+^) (4), 109 (100). ^1^H NMR (400 MHz, DMSO-d6, 293 K): δ = 2.12 (s, 3H, CH_3_), 5.04−5.16 (m, 3H, C*H_2_*C*H*N), 6.91 (t, 2H, *J* = 7.6 Hz, ArH), 7.03 (t, 2H, *J* = 7.6 Hz, ArH), 7.31−7.35 (m, 4H, ArH), 7.54 (d, 2H, *J* = 8.0 Hz, ArH), 7.68 (s, 1H, ArH), 10.86 (brs, 2H, NH); ^13^C NMR (100 MHz, DMSO-d6, 293 K): δ = 10.9, 35.5, 53.9, 111.9, 115.3, 118.7, 119.2, 121.4, 122.8, 123.2, 126.8, 136.8, 141.7.

### 2.3. Bacterial Strains

Six reference human pathogens were used in this study, *Escherichia coli* ATCC 35218, *Pseudomonas aeruginosa* ATCC 9027, *Staphylococcus aureus* ATCC 43387, *Enterococcus faecalis* ATCC 29212, and *Candida albicans* ATCC 14053. The clinical human strain *Klebsiella pneumoniae* 6/4, isolated from patient with urinary infection and kindly provided by Gamma Laboratory (Fano, Pesaro, Italy), was also included. 

All of the strains were maintained in Tryptic Soy Agar (TSA, Oxoid, Milan, Italy) at 37 °C, while *C. albicans* ATCC 14053 was grown in Sabouraud Dextrose Agar (Oxoid, Milan, Italy). All the stock cultures were kept at −80 °C in Nutrient broth (Oxoid, Milan, Italy) with 15% of glycerol.

### 2.4. Determination of Minimum Inhibitory Concentration (MIC)

MICs were determined by standard micro-dilution method. First, each compound was dissolved in dimethyl sulfoxide (DMSO) (Sigma, Milan, Italy) of biological grade. Several colonies of each bacterial strain were inoculated in 10 mL of sterile Mueller-Hinton broth (MHB) (Oxoid) and incubated at 37 °C for 18–24 h. Each bacterial suspension was adjusted to about 10^6^ cfu/mL (OD_610nm_ 0.13–0.15) and 100 µL was added in wells of the 96-well cell culture plate (Cellstar^®^, Greiner Bio-One, Frickenhausen, Germany), together with the appropriate volumes of the test solutions (from 0.5 to 128 µg/mL). Two rows were used for positive (bacteria alone) and negative controls (MHB alone), respectively. Gentamicin (from 0.125 to 128 µg/mL) and fluconazole (Sigma, Milan, Italy) were also added as internal controls. Preliminary assays with DMSO were carried out to exclude its possible bacteriostatic and/or bactericidal activity; the volume of DMSO added in each well never exceeded 5% (*v/v*) of the final total volume. The optical density (600 nm) of each well was assessed using a Multiscan Ex Microplate Reader (Thermo Scientific, Italy). MIC was defined as the lowest concentration of compound inhibiting the bacterial growth after 24 h of incubation. All data were expressed as the mean of three independent experiments that were performed in duplicate.

### 2.5. Crystal Violet Biofilm Assay 

A static biofilm formation assay was performed in 24-wells polystyrene plates (VWR, Milan, Italy) and biomass production was assessed after Cristal Violet (CV) staining, as described in Campana et al. [24]. All of the strains were grown in Tryptic Soy Broth (TSB, Oxoid, Milan, Italy) at 37 °C for 24 h to obtain a bacterial suspension at the end of the logarithmic phase. Subsequently, the optical density of each suspension (OD _610 nm_) was adjusted to about 0.13–0.15 (correspondent to 10^6^–10^7^ cfu/mL); 1 mL of each suspension, diluted 1:10 in TSB, was seeded in 24-well polystyrene plates (Cellstar^®^, Greiner Bio-One, Frickenhausen, Germany), and incubated at 37 °C for 24 h to allow biofilm formation. At the end of incubation, the wells were once washed with PBS to eliminate unattached cells and covered with crystal violet (CV) 0.1% (*v/v*) for 15 min. The samples were once washed again with PBS and air-dried. The remaining CV was dissolved in 85% ethanol (15 min at room temperature) and, finally, 200 µL from each well was transferred to a 96-well cell culture plate (Cellstar^®^, Greiner Bio-One, Frickenhausen, Germany) for spectrophotometry at 570 nm, using a Multiscan Ex Microplate Reader (Thermo Scientific). Each data point was averaged from at least eight replicate wells. The experiments were performed twice using independent cultures.

### 2.6. Biofilm Formation Inhibition

The anti-biofilm activity of each compound against each pathogen was determined in terms of biofilm formation inhibition. Briefly, 200 µL of each bacterial suspension (about 10^6^ bacteria/mL) were inoculated in 24-well polystyrene plates (Cellstar^®^, Greiner Bio-One, Frickenhausen, Germany) with the corresponding amount of each selected compound at their relative MIC and 2× MIC values. Two wells for each pathogen were also inoculated with bacteria in Tryptic Soy Broth (TSB) (Oxoid, Milan Italy) and included as controls. The plates were then incubated for 24 h at 37 °C to allow biofilm development; at the end of the incubation, each sample was gently once washed by PBS and the biofilm formation inhibition for each pathogen was assessed by CV staining. All data were expressed as the mean of three independent experiments performed in duplicate.

### 2.7. Eradication of Biofilms with Selected Chemical Compounds

In these last experiments, the activity of selected compounds (**1**, **3**, **4**, and **8**) on developed biofilms of *E. coli* ATCC 35218, *S. aureus* ATCC 43387, *K. pneumoniae* 6/4, and *C. albicans* ATCC 10231 was determined. Briefly, biofilms of each pathogen were prepared with the procedure described above (Crystal violet biofilm assay section). After 24 h of incubation at 37 °C, all of the biofilms were gently once washed with PBS and covered for 30 min with the right amount of each compound corresponding to the MIC. For each plate, two wells were treated with physiological saline (negative controls). After antimicrobial treatment, all of the biofilms were once washed by PBS and adherent bacteria were harvested by sterile scrapers in 1 mL of sterile physiological saline solution, subsequently serially diluted in the same medium and plated in triplicate on TSA (Oxoid, Milan, Italy). The plates were then incubated at 37 °C for 24 h at the end of which the colony forming units (cfu) were enumerated. To verify the eventual reduction of biomass production after antimicrobial treatment, CV staining was carried out as described above. All the data were expressed as the mean of three independent experiments performed in duplicate.

### 2.8. Statistical Analysis

Statistical analysis was performed using Prism version 5.0 (GraphPad Inc., La Jolla, CA, USA). The assumptions for parametric test were cheeked prior to carry out the statistical analysis by *t*-Student test. *p* values < 0.05 were considered to be statistically significant.

## 3. Results

### 3.1. Determination of Minimum Inhibitory Concentration 

The compound **1** showed the greatest antimicrobial activity against all the pathogens included in this study. In detail, the lowest MIC value of 8 µg/mL was observed for *E. coli* ATCC 35218, *S. aureus* ATCC 43387, and *K. pneumoniae* 6/4, while higher values were determined for *E. faecalis* ATCC 29212 (32 µg/mL), *P. aeruginosa* ATCC 9027, and *C. albicans* ATCC 10231 (64 µg/mL) (Table 1). As regards compound **3**, the lowest MIC value (64 µg/mL) was evidenced for *E. coli* ATCC 35218 and 128 µg/mL for all others species. Compound **4** showed 16 µg/mL MIC value toward *E. coli* ATCC 35218 and 32 µg/mL against *K. pneumoniae* 6/4 and *C. albicans* ATCC 10231. MIC values of 128 µg/mL were registered for compound **8**, while all the other compounds showed MICs >128 µg/mL for all the tested microorganisms. With regard to the internal control, gentamicin inhibited microbial growth with the lowest MIC value of 8 µg/mL for *K. pneumoniae* 6/4 and the highest MIC value of 64 µg/mL for *E. faecalis* ATCC 29212 (Table 1). *C. albicans* ATCC 10231 resulted in being sensitive to fluconazole with MIC of 1 µg/mL.

### 3.2. Biofilm Formation Inhibition

The compounds **1**, **3**, **4**, and **8** were tested at the relative MIC and 2× MIC values during the biofilm formation of the examined pathogens (Table 2). In many cases, the compounds at their relative MIC and 2× MIC values determined the bactericidal effect after 24 h of incubation (no visible growth in the wells) and, in these cases biofilm formation after CV staining were not assessed (Figure S1). *E. faecalis* ATCC 29212 and *P. aeruginosa* ATCC 9027 were not included on the basis of the results that were obtained during the biofilm formation, where *E. faecalis* resulted in being completely inhibited by all the tested compounds (bactericidal effect) while *P. aeruginosa* was resistant.

As general trend, compound **1** was able to reduce biofilm formation of most the examined bacteria at their relative MIC values, with the only exception of *P. aeruginosa* ATCC 9027. The lowest concentration (8 µg/mL), tested against *E. coli* ATCC 35218 and *S. aureus* ATCC 43387, provoked 68.3 and 82.2% of biofilm formation inhibition, respectively; the same concentration induced only 40.0% of *K. pneumoniae* 6/4 biofilm formation inhibition, reaching 83.7% of inhibition with 16 µg/mL (2× MIC value). The highest concentration (64 µg/mL) was unable to inhibit the biofilm formation of *P. aeruginosa* ATCC 9027, while it exhibited bactericidal action against *C. albicans* ATCC 10231. The compound **3** (MIC 64 µg/mL) was able to strongly inhibit the biofilm formation of *E. coli* ATCC 35218 (97.6%). The highest MIC value (128 µg/mL) determined only 34.6% of biofilm formation inhibition in *C. albicans* ATCC 10231 and it was unable to contrast the biofilm formation of *P. aeruginosa* ATCC 9027 (7.8%). On the other hand, 128 µg/mL resulted in being bactericidal against *S. aureus* ATCC 43387, *E. faecalis* ATCC 29212, and *K. pneumoniae* 6/4. The compound **4** was tested at MIC values ranging from 16 to 128 µg/mL. The lowest concentration was bactericidal against *E. faecalis* ATCC 29212, while the biofilm formation of *K. pneumoniae* 6/4 was remarkably inhibited (64.7%) by MIC value of 32 µg/mL, reaching 96.6% of inhibition with 64 µg/mL (2× MIC value). Less biofilm inhibition (14.6%) was observed for *C. albicans* ATCC 10231, with 32 µg/mL, and also with 2× MIC value (40.9%). The highest concentration of compound **4** (128 µg/mL) caused 56.6% of *S. aureus* ATCC 43387 biofilm formation inhibition and resulted in being bactericidal against *E. faecalis* ATCC 29212. As regards compound **8** (MIC 128 µg/mL), remarkable biofilm formation inhibition was evidenced for *K. pneumoniae* 6/4 (65.8%), while lower percentages were observed for *E. coli* ATCC 35218 (54.1%) and *S. aureus* ATCC 43387 (34.9%); also, in this case, the biofilm formation of *P. aeruginosa* ATCC 9027 was not reduced, while a bactericidal effect was observed against *E. faecalis* ATCC 29212 and *C. albicans* ATCC 10231. Increased percentages of biofilm formation inhibition were always observed with compound **8** at 2× MIC value.

### 3.3. Eradication of Biofilms after Treatment with Compounds ***1***, ***3***, ***4*** and ***8***

**Figure 6:**
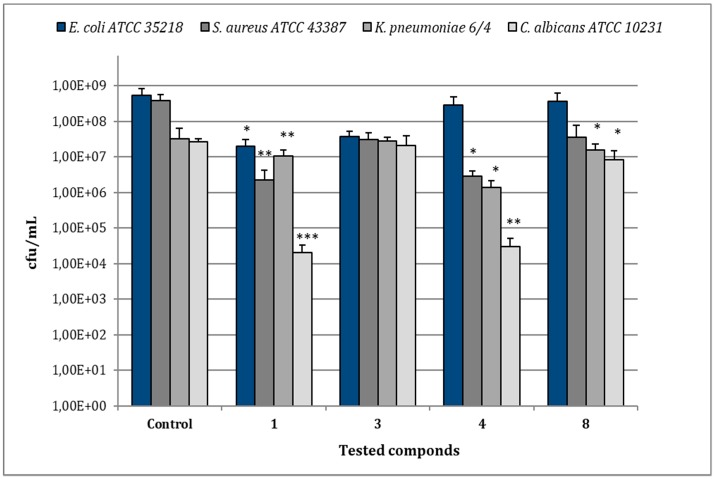
Assessment of viable cells harvested from biofilms of *E. coli* ATCC 35218, *S. aureus* ATCC 43387, *K. pneumoniae* 6/4 and *C. albicans* ATCC 10231 after 30 min of treatment with compounds **1**, **3**, **4**, and **8** at their relative MIC values. Data represent the mean ± SD of cfu/mL values obtained in three independent experiments performed in duplicate. Asterisks represent values statistically significant (* *p* < 0.05; ** *p* < 0.01; *** *p* < 0.001) as compared to the related controls.

In the case of *E. coli* ATCC 35218, the treatment of biofilms with compound **1** (MIC 8 µg/mL) reduced bacterial viability to 2.0 × 10^7^ cfu/mL in comparison to 5.28 × 10^8^ cfu/mL of the relative control biofilm (*p* < 0.05), while the other compounds caused no significant reduction of cfu/mL (Figure 6). For *S. aureus* ATCC 43387, compound **1** (MIC 8 µg/mL) reduced viable bacteria to 2.27 × 10^6^ cfu/mL in comparison to 3.85 × 10^8^ cfu/mL of the untreated control (*p* < 0.001) and a statistical significant reduction in cfu/mL values was also observed after treatment with compounds **4** (MIC 128 µg/mL) (*p* < 0.01). As regards *K. pneumoniae* 6/4, the treatment with compounds **1** (MIC 8 µg/mL) and **4** (MIC 32 µg/mL) determined values of 1.0 × 10^7^ and 1.37 × 10^6^ cfu/mL, respectively, with a significant decrease in comparison to the relative control biofilm (3.2 × 10^7^ cfu/mL) (*p* < 0.01; *p* < 0.05). No remarkable reduction of *K. pneumoniae* 6/4 growth was observed with compounds **3** and **8** (MIC 128 µg/mL). In the case of *C. albicans* ATCC 10231, the most remarkable decrease of cfu/mL was observed after the treatment with compound **1** (MIC 64 µg/mL), showing 2.0 × 10^4^ cfu/mL in comparison to 2.6 × 10^7^ cfu/mL of the control one (*p* < 0.001). Similarly, the treatment with compounds **4** (MIC 32 µg/mL) evidenced 3.0 × 10^4^ cfu/mL (*p* < 0.01), while compounds **3** and **8** (MICs 128 µg/mL) induced only a slight reduction.

**Figure 7:**
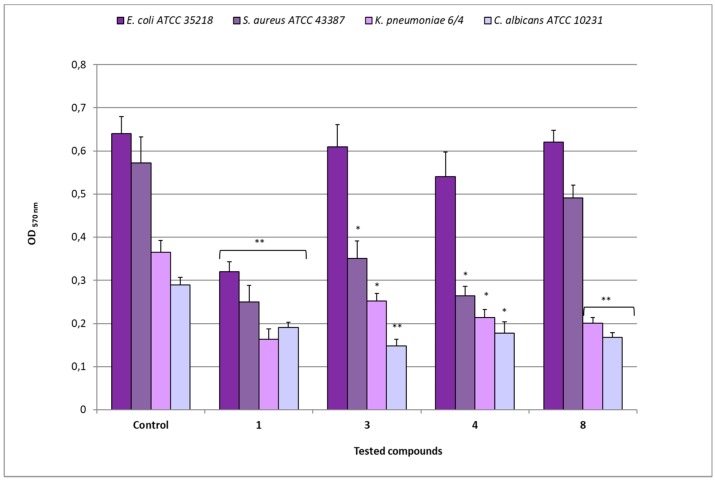
Disaggregating efficacy of compounds **1**, **3**, **4**, and **8** at their relative MIC values, against biofilms of *E. coli* ATCC 35218, *S. aureus* ATCC 43387, *K. pneumoniae* 6/4 and *C. albicans* ATCC 10231, as assessed by spectrophotometer reader (OD _570nm_) after 30 min of contact. Data represent the mean ± SD obtained in three independent experiments performed in duplicate. Asterisks represent values statistically significant (* *p* < 0.05; ** *p* < 0.01) as compared to the related controls.

The biomass analysis revealed different decreases of OD _570 nm_ values in biofilms after treatment with the different compounds (Figure 7). As regards *E. coli* ATCC 35218, after exposure to compound **1** (MIC 8 µg/mL), an OD of 0.320 was observed in comparison to 0.641 of the relative untreated biofilm (*p* < 0.01), while the treatment with compounds **3** (MIC 64 µg/mL), **4** (MIC 16 µg/mL), and **8** (MIC 128 µg/mL) showed OD values that were quite similar (from 0.541 to 0.622) to that of the control. The exposure of *S. aureus* ATCC 43387 biofilms to compound **1** (MIC 8 µg/mL) determined an OD of 0.250 in comparison to 0.573 of the relative untreated biofilm (*p* < 0.01), while after treatment with compounds **3** and **4** (MICs 128 µg/mL), OD values of 0.350 and 0.264, respectively (*p* < 0.05) were registered. On the contrary, compound **8** (MIC 128 µg/mL) determined an OD value quite similar to that of the relative control. 

For *K. pneumoniae* 6/4, compound **1** (MIC 8 µg/mL) determined an OD of 0.163 in comparison to 0.365 of the relative untreated biofilm (*p* < 0.01), while the treatment with compounds **3** (MIC 128 µg/mL), **4** (MIC 32 µg/mL), and **8** (MIC 128 µg/mL) induced 0.252, 0.214 (*p* > 0.05), and 0.201 (*p* < 0.01) OD values, respectively. In the case of *C. albicans* ATCC 10231, the treatment with compound **1** (MIC 64 µg/mL) reduced the biomass to OD 0.191 in comparison to 0.290 of the control one (*p* < 0.01); similarly, after exposure to compounds **3** (MIC 128 µg/mL), **4** (MIC 32 µg/mL), and **8** (MIC 128 µg/mL), OD values of 0.148 (*p* < 0.01), 0.178 (*p* < 0.05) and 0.168 (*p* < 0.01), respectively, were registered.

In Table 3 are summarized the percentages of biomass reduction, index of eradication activity, induced by each compound against the tested developed biofilms. As shown, after 30 min of contact with compound **1**, reductions greater than 50% were observed for most of the examined strains, with the exception of *C. albicans* ATCC 10231 (only 34.2%). On the contrary, all the derivatives (**3**, **4**, and **8**) showed low activity against *E. coli* ATCC 35218 biofilms, in some cases, also the derivatives reached remarkable percentages of biomass reduction. Interestingly, compound **3** reduced biofilm of *C. albicans* ATCC 10231 (48.8%), resulting the most active compound against mycete biofilms. Compound **4** showed strong anti-biofilm activity against *S. aureus* ATCC 43387 with percentage comparable to compound **1** (53.9% vs 56.3%), while compound **8** acted as good disaggregating agent on *K. pneumoniae* 6/4 (44.9%) and *C. albicans* ATCC 10231 (41.9%) biofilms.

## 4. Discussion

Currently, the research is being conducted to discover novel compounds that are able to inhibit microbial biofilms, without allowing bacteria to develop drug resistance. Marine invertebrates have been proved to be a rich source of bioactive compounds with different ecological functions [6,25]. Among these, sponges are marine sessile filters exposed to large amounts of bacteria in the surrounding seawater and their potential antimicrobial activity attracted the attention of many researchers [10,26,27].

In the present study, the antimicrobial activity of **1** and its synthetic derivatives **2**–**8** was investigated (Table 1). The synthesis of heterocyclic compounds **1**–**4** and **6**–**8** was carried out according to literature procedures [16,18]. Regarding the preliminary SAR, our data evidenced that MICs of compound **1** resulted in being lower (from 8 to 64 µg/mL) as compared to those of its derivatives (from 16 to 128 µg/mL) and, in some cases, lower than the antibiotic gentamicin (*E. coli*, *S. aureus*, and *E. faecalis*). Moreover, the MIC of **1** for *S. aureus* is comparable with those of tulongicin and compound **11** (Figure 1) [22,23]. The effect of the chemical modification of **1** was then studied, all the derivatives **2**–**8** also share a DIM molecular unit with all the compounds reported in Figure 1. Removing the bromine atoms (compound **2**) or the ethylamine side chain (compound **5**), resulted in being detrimental for the antimicrobial activity with MICs greater than 128 µg/mL for all the tested microorganisms. On the contrary, the substitution of the bromine atoms with fluorines or the methylation of NHs (compounds **3** and **4**, respectively), maintained the antimicrobial activity of the compounds against the tested microorganisms, even if with MICs values that are higher when compared to those of compound **1**. To study the influence of an additional *N*-based heterocycle on the antimicrobial activity [22,28], an ethyl-azole side chain was introduced (compounds **6**–**8**). Compounds **6** and **7**, presenting an ethyl-pyrrole and -imidazole decoration, respectively, were not active as antimicrobial agents (MICs greater than 128 µg/mL), while the bisindole-triazole conjugate **8** evidenced a moderate antimicrobial activity (MIC 128 µg/mL). No significant difference in the antimicrobial activity of all the tested compounds emerged between gram positive and gram negative tested bacteria.

Subsequently, on the basis of their antimicrobial activity, the most active bisindoles were also investigated as an inhibitor of bacterial biofilm formation. It is well-known that bacteria organized in biofilm are more resistant to antimicrobials because the stratification of biofilm itself and the auto-produced matrix protect them from the action of antibiotics or disinfectants [29,30]. In our experiments, the lead compound 1 exhibited anti-biofilm activity against all the examined bacteria (biofilm formation inhibition greater than 50%), at its MICs values, with the exception of *K. pneumoniae* 6/4 (only 40%). When tested at 2× MIC values, **1** resulted to be bactericidal against all the microorganisms, reaching 83.7% of biofilm formation inhibition in the case of *K. pneumoniae* 6/4. Interestingly, no inhibition was observed with 1 toward *P. aeruginosa* ATCC 9027 biofilm, both at MIC and 2× MIC values, pointing out the well-known resistance of this microorganism to antimicrobials [31,32]. The selected derivatives (**3**, **4** and **8**) were also able to inhibit the biofilm formation of most of the examined microorganisms at their MIC values, with the exclusion of *P. aeruginosa* ATCC 9027. In detail, compound **3** (fluorine atoms instead of bromines) showed high anti-biofilm activity against *E. coli* ATCC 35218, reaching 97.6% of biofilm formation inhibition at 64 µg/mL MIC value, but it was less active against *C. albicans* ATCC 10231 (34.6% of inhibition at 128 µg/mL). Similarly, compounds **4** (NHs methylated) and **8** (bisindole-triazole conjugate) were active against *K. pneumoniae* 6/4, with percentages of biofilm formation inhibition of 64.7% (at 64 µg/mL) and 65.8% (at 128 µg/mL), respectively (Table 2). In any case, all the synthetic derivatives (**3**, **4**, and **8**) were active at MIC values higher when compared to those of the lead compound, thus compound **1** resulted in the best performing one.

After determining the efficacy of our selected compounds on microbial biofilms formation, their eradication activity against already developed biofilms was assessed. In this case, the molecules were added for 30 minutes to different biofilms in order to quantify their disaggregating activity. Our findings evidenced that **1** significantly eradicated all the tested biofilms, while **3**, **4**, and **8** showed a variable degree of disaggregating activities dependent on the bacterial species. It can be noted that the biofilm disaggregating activity was not tightly correlated with the observed anti-bacterial activity (as decrease of cell viability) against the examined microbial targets. Regarding the SAR, the chemical modifications of compound **1** did not improve disaggregating property against *E. coli* ATCC 35218 biofilms. In the case of *S. aureus* ATCC 43387 biofilms, the NHs methylation (compound **4**) did not alter the anti-bioflm activity, which resulted in being similar to that of **1**, while the other decorations originated compounds that were less active. Regarding biofilms formed by *C. albicans* ATCC 10231, the most active disaggregating agent resulted **3** presenting fluorines instead of bromines. Compound **8**, having triazole moiety, showed similar anti-biofilm activity when compared to **1** against *K. pneumoniae* 6/4 and *C. albicans* ATCC 10231 biofilms. It can be observed that the presence of bromine and ethylamine sidechain is essential for the antibiofilm activity of examined compounds, as also reported by Bunders et al. [33] for similar compounds (flustramine derivatives) against *E. coli* biofilm. It is well-known that indole signaling is involved in the regulation of a number of bacterial behaviors, including antibiotic resistance, virulence, and biofilm formation [34]. In this study, the marine alkaloid **1** and its derivatives contain two units of indole, thus suggesting that the observed antibiofilm activity may result from a modulation of indole-based signaling pathways.

## 5. Conclusions 

In conclusion, our data highlight the potential of the natural marine alkaloid 2,2-bis(6-bromo-3-indolyl) ethylamine **1** as antimicrobial and anti-biofilm agent. The modifications of the lead compound **1** have evidenced that the presence of halogens and/or ethylamine side chain are important for the maintenance of both antimicrobial and anti-biofilm activities. On the contrary, the addition of azole moiety to **1** was not effective in terms of microbial biofilms interaction. To our knowledge, this is the first work reporting the anti-biofilm activity of a natural bisindole against a broad spectrum of microorganisms. Compound **1** has shown MIC values that are comparable with those of similar compounds sharing the DIM molecular unit. However, the compounds that were reported in this work were prepared following a safe, straightforward, and scalable synthesis, resulting in being cheaper than others of the same category [19,20,21,22,23]. Therefore, compound **1** and some of its derivatives could be proposed for sanitation applications of environmental surfaces (such as industrial equipment, abattoirs, processing surfaces). Further studies will be necessary to assess the molecular mechanism of the active compounds and their efficacy against bacterial biofilms that formed on different surfaces and against mixed-species biofilms.

## Figures and Tables

**Table 1:** Minimum Inhibitory Concentration (MIC) values (µg/mL) of the tested compounds against selected microbial strains, assessed by broth microdilution method according to the Performance Standards for Antimicrobial Susceptibility Testing (CLSI Document M100-S2, 2013). Gentamicin and fluconazole were used as internal controls.

Microbial Strains	1	2	3	4	5	6	7	8	Gentamicin
*E. coli* ATCC 35218	8	>128	64	16	>128	>128	>128	128	16
*S. aureus* ATCC 43387	8	>128	128	128	>128	>128	>128	128	16
*E. faecalis* ATCC 29212	32	>128	128	128	>128	>128	>128	128	64
*P. aeruginosa* ATCC 9027	64	>128	128	128	>128	>128	>128	128	16
*K. pneumoniae* 6/4	8	>128	128	32	>128	>128	>128	128	8
*C. albicans* ATCC 10231	64	>128	128	32	>128	>128	>128	128	1 ^a^

^a^ Fluconazole was used as control for *C. albicans* ATCC 10231.

**Table 2:** Biofilm formation inhibition exerted by compounds **1**, **3**, **4**, and **8**. Each molecule was added at its relative MIC and 2× MIC (µg/mL) values during the biofilm formation of the different pathogens; the inhibition percentages were determined after spectrophotometer reader at 570 nm.

Microbial Strains	1			3			4			8		
		Biofilm Inhibition			Biofilm Inhibition			Biofilm Inhibition			Biofilm Inhibition	
	MIC	MIC	2× MIC	MIC	MIC	2× MIC	MIC	MIC	2× MIC	MIC	MIC	2× MIC
*E. coli* ATCC 35218	8	68.3%	BE	64	97.6%	BE	16	1.7%	48.1%	128	54.1%	92.0%
*S. aureus* ATCC 43387	8	82.2%	BE	128	BE	ND	128	56.6%	BE	128	34.9%	62.9%
*E. faecalis* ATCC 29212	32	BE	ND	128	BE	ND	128	BE	ND	128	BE	ND
*P. aeruginosa* ATCC 9027	64	0.5%	0.9%	128	7.8%	BE	128	4.8%	BE	128	7.1%	BE
*K. pneumoniae* 6/4	8	40.0%	83.7%	128	BE	ND	32	64.7%	96.6%	128	65.8%	98.7%
*C. albicans* ATCC 10231	64	BE	ND	128	34.6%	BE	32	14.6%	40.9%	128	BE	ND

BE, bactericidal effect: biomass was not determined by spectrophotometer reader (570 nm) because no bacterial growth was visible. in the wells (see also Figure S1); ND: not determined because already bactericidal at MIC value.

**Table 3:** Percentages of biofilm eradication activity assessed for *E. coli* ATCC 35218, *S. aureus* ATCC 43387, *K. pneumoniae* 6/4 and *C. albicans* ATCC 10231 after 30 min of contact with compounds **1**, **3**, **4**, and **8** at their relative MIC (µg/mL) values. Percentages were determined after spectrophotometer reader (OD _570nm_).

Preformed Biofilms	1	3	4	8
*E. coli* ATCC 35218	50.0%	4.7%	15.7%	3.1%
*S. aureus* ATCC 43387	56.3%	38.9%	53.9%	14.2%
*K. pneumoniae* 6/4	55.3%	30.9%	41.3%	44.9%
*C. albicans* ATCC 10231	34.2%	48.8%	38.6%	41.9%

## Supplementary Materials

The following are available online at http://www.mdpi.com/2076-2607/7/2/28/s1.
