# Supplementary material for: Marine Alkaloid 2,2-Bis(6-bromo-3-indolyl) Ethylamine and Its Synthetic Derivatives Inhibit Microbial Biofilms Formation and Disaggregate Developed Biofilms
Source: Microorganisms. 2019 Jan 23;7(2):28. doi: 10.3390/microorganisms7020028 (PMC6406822; doi:10.3390/microorganisms7020028)
Supplement: Supplementary file 1 [file microorganisms-07-00028-s001.pdf]

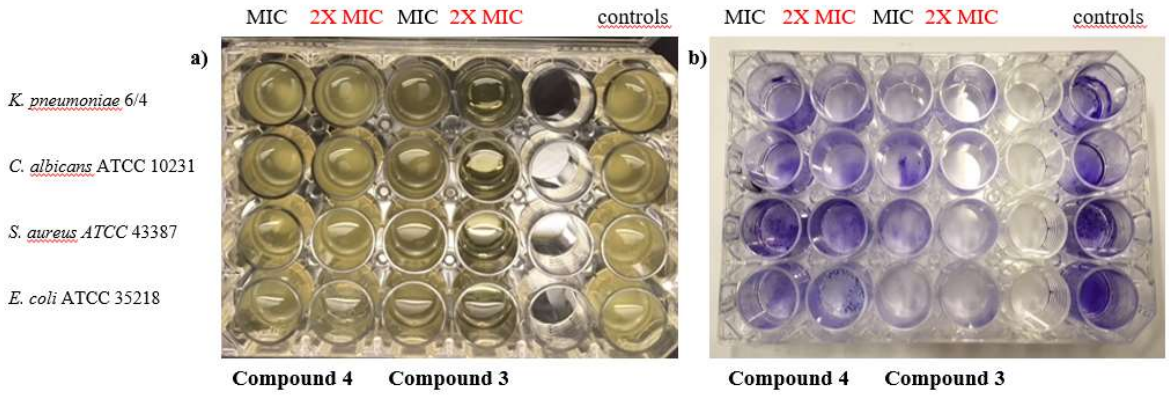

Figure S1. Biofilms formation of *K. pneumoniae* 6/4, *C. albicans* ATCC 10231, *S. aureus* ATCC 43387 and *E. coli* ATCC 35218 in presence of compounds **3** and **4** at their relative MIC and 2× MIC values after 24 h of incubation at 37 °C (**a**). Biofilms formation inhibition was assessed by CV staining. Representative images of biofilms formation inhibition (**b**) as appear after PBS washing and prior to CV ethanol dissolution and reading at 570 nm: the clear wells with no formed biofilms were impossible to read by spectrophotometer because the staining resulted negative (complete inhibition).
